# Exploring Natural Products with Antioxidant and Anticancer Properties

**DOI:** 10.3390/ph19060963

**Published:** 2026-06-22

**Authors:** Sikiru Olaitan Balogun, Edson Lucas dos Santos

**Affiliations:** 1 Research Group on Biotechnology and Bioprospecting Applied to Metabolism and Cancer (GEBBAM), Universidade Federal da Grande Dourados, Dourados-Itahum Highway, Km 12, Dourados 79804-970, MS, Brazil; edsonsantosphd@gmail.com; 2 Programa de Pós-Graduação em Ciências da Saúde, Faculdade de Ciências da Saúde, Universidade Federal da Grande Dourados, Dourados 79804-970, MS, Brazil; 3 Faculdade do Vale do Juruena (Faculdade AJES), Avenida Gabriel Muller 1086 N, Modulo 01, Juína 78320-000, MT, Brazil

Natural products continue to play a defining role in the discovery of anticancer agents. Several of the most important chemotherapeutic drugs in current clinical use, including paclitaxel, the vinca alkaloids, and camptothecin derivatives, were derived from or inspired by natural sources, demonstrating the continued relevance of natural compounds to oncology [1]. This longstanding contribution is not surprising considering their structural complexity and their multi-target capacity, both of which are essential in diseases where oxidative stress, inflammation, metabolic dysfunction, and oncogenic signaling interact in nonlinear ways. As research increasingly integrates redox biology with cancer pathophysiology, compounds with both antioxidant and anticancer actions have attracted significant attention.

This Special Issue of Pharmaceuticals presents a collection of studies that capture this scientific moment. A clear trend across the included papers is the movement toward mechanistic clarity, analytical rigor, and translational intent. Rather than focusing solely on cytotoxicity or antioxidant measurements, the contributions emphasize molecular targets, pathway interactions, and validated experimental frameworks. This shift is necessary to ensure that natural compounds are evaluated not only for their bioactivity but also for their pharmacological plausibility.

One of the strongest developments in the field is the increased use of metabolomics in natural-product-based cancer research. Dabbousy et al. [2] demonstrate how metabolomics can identify bioactive metabolites, reveal metabolite–pathway relationships, and strengthen quality control. Their work emphasizes essential steps such as authenticated botanical sourcing, validated extraction procedures, optimized storage conditions, and the integration of MS and NMR technologies. Through these improvements, metabolomics helps address longstanding concerns about extract variability and mechanistic uncertainty.

Secondary metabolites remain central to anticancer drug discovery, and several papers in this Special Issue highlight their roles. Compounds such as flavonoids, terpenoids, alkaloids, lignans, and tannins influence key biological processes including apoptosis, angiogenesis, mitochondrial remodeling, cell-cycle regulation, and redox homeostasis. Siddiqui et al. [3] used network pharmacology and experimental validation to investigate *Asparagus racemosus* in triple-negative breast cancer, identifying functional pathways and molecular targets connected to proliferation and survival. Cakir et al. [4] examined *Ficus carica* latex in HPV-positive cervical cancer and found alterations in cell-cycle gene expression, reinforcing the relevance of traditional medicinal materials when studied with modern molecular tools.

Polyphenols, reviewed by El Oirdi [5], represent another important category. Evidence from clinical, animal, and in vitro studies demonstrates their roles in modulating oxidative stress, inflammation, metabolic pathways, and immune responses. These characteristics make polyphenols attractive candidates for chemoprevention. Jain et al. [6] further describe how dietary polyphenols support antioxidant capacity and metabolic health. Chunarkar-Patil et al. [7] expand this discussion by mapping natural molecules, including polyphenols, into computational pipelines that accelerate the search for anticancer candidates.

Promising activity, however, does not guarantee therapeutic effects. One of the major barriers to translation remains poor bioavailability. Many natural compounds suffer from low solubility, rapid metabolism, and limited absorption, resulting in weak in vivo performance despite strong in vitro findings. Patel et al. [8] discuss nanotechnology-based delivery systems that can improve the solubility, stability, and targeted delivery of anticancer molecules. Similar concerns and solutions appear in recent clinical and experimental reports, including studies focusing on plant- and marine-derived compounds in prostate cancer [9] and mechanisms regulating treatment response [10].

Variability in natural preparations also remains a challenge. Chemical composition can vary due to geography, climate, soil composition, plant age, extraction technique, and storage conditions. Such variability limits reproducibility and complicates regulatory evaluation. Pirintsos et al. [10] argue that integrating ethnopharmacological knowledge with modern analytical methods can enhance reliability by identifying consistent phytochemical patterns and validating traditional claims using robust scientific frameworks. This model, alongside digital and network-pharmacology strategies such as those applied by Arif et al. [11] in their study of *Nigella sativa* for breast cancer, supports a more reproducible and mechanistically grounded approach to natural drug discovery.

To make meaningful progress, natural-product-centered oncology must increasingly integrate multi-omics. Genomics, transcriptomics, proteomics, and metabolomics, when combined, allow researchers to map target networks, identify metabolic vulnerabilities, and connect specific compounds to their mechanistic profiles. The contributions in this Special Issue illustrate how this integration can be implemented effectively. Studies on marine fungi, algae, cyanobacteria, and invertebrates reveal the chemical novelty that exists beyond terrestrial plants and underscore the importance of expanding biodiscovery efforts to underexplored ecosystems.

Taken together, the papers in this Special Issue emphasize that natural products remain promising sources of antioxidant and anticancer agents, but that their development requires methodological rigor, mechanistic precision, and translational focus. Enhancing pharmacokinetics, improving delivery strategies, standardizing natural preparations, and designing robust clinical studies should be priorities for future work. Researchers should continue to incorporate rigorous computational, molecular, and analytical approaches, as demonstrated by the studies in this edition, to advance the field toward clinically viable applications.

The Guest Editors thank all authors, reviewers, and collaborators for their contributions. This Special Issue provides a strong foundation for the next steps in natural product pharmacology and reinforces the importance of integrating mechanistic, analytical, and translational work to unlock the therapeutic potential of natural compounds.
